# Lights and shadows on the use of metformin in pregnancy: from the preconception phase to breastfeeding and beyond

**DOI:** 10.3389/fendo.2023.1176623

**Published:** 2023-06-20

**Authors:** Giulia Tosti, Annarita Barberio, Linda Tartaglione, Alessandro Rizzi, Mauro Di Leo, Luca Viti, Angelo Sirico, Sara De Carolis, Alfredo Pontecorvi, Antonio Lanzone, Dario Pitocco

**Affiliations:** ^1^ Diabetes Care Unit, Fondazione Policlinico Universitario A. Gemelli Istituto di Ricerca e Cura a Carattere Scientifico (IRCCS), Rome, Italy; ^2^ Catholic University School of Medicine, Rome, Italy; ^3^ Department of Woman and Child Health, Woman Health Area Fondazione Policlinico Universitario A. Gemelli Istituto di Ricerca e Cura a Carattere Scientifico (IRCCS), Rome, Italy; ^4^ Department of Endocrinology, Fondazione Policlinico Universitario A. Gemelli Istituto di Ricerca e Cura a Carattere Scientifico (IRCCS), Rome, Italy

**Keywords:** gestational diabetes, insulin resistance, pregnancy, metformin, offspring, fertilization

## Abstract

During pregnancy, the complex hormonal changes lead to a progressive decrease of insulin sensitivity that can drive the onset of gestational diabetes (GDM) or worsen an already-known condition of insulin resistance like type 2 diabetes, polycystic ovarian syndrome (PCOS), and obesity, with complications for the mother and the fetus. Metformin during pregnancy is proving to be safe in a growing number of studies, although it freely crosses the placenta, leading to a fetal level similar to maternal concentration. The aim of this literature review is to analyze the main available evidence on the use of metformin during, throughout, and beyond pregnancy, including fertilization, lactation, and medium-term effects on offspring. Analyzed studies support the safety and efficacy of metformin during pregnancy. In pregnant women with GDM and type 2 diabetes, metformin improves obstetric and perinatal outcomes. There is no evidence that it prevents GDM in women with pregestational insulin resistance or improves lipid profile and risk of GDM in pregnant women with PCOS or obesity. Metformin could have a role in reducing the risk of preeclampsia in pregnant women with severe obesity, the risk of late miscarriages and preterm delivery in women with PCOS, and the risk of ovarian hyperstimulation syndrome, increasing the clinical pregnancy rate in women with PCOS undergoing *in vitro* fertilization (IVF/FIVET). Offspring of mothers with GDM exposed to metformin have no significant differences in body composition compared with insulin treatment, while it appears to be protective for metabolic and cardiovascular risk.

## Introduction

During pregnancy, the human body faces complex hormonal changes leading to a physiologic progressive decrease of insulin sensitivity (1). The physiologic factors responsible for the decrease of insulin sensitivity or insulin resistance of pregnancy are not completely understood, but they are partially related to the metabolic effects of maternal plasma progesterone, human placental lactogen, free cortisol, and estrogens that are elevated in the maternal circulation during pregnancy (2).

The decrease in insulin sensitivity in physiological pregnancy leads to higher glucose output and lower glucose uptake and utilization with the purpose to ensure fetal energy requirements (3).

In some cases, the imbalance of these metabolic changes can lead to the onset of gestational diabetes (GDM), which can manifest itself according to three different phenotypes: fasting hyperglycemia, postprandial hyperglycemia, and mixed hyperglycemia (4).

In some other cases, the physiological reduction of insulin sensitivity is established on an initial picture already characterized by insulin resistance as in the case of pregnancies that occur in obese patients, patients with polycystic ovarian syndrome (PCOS), and patients with already diagnosed type 2 diabetes (T2DM) (5–7).

The unbalanced insulin resistance that is developed in all these pathologies during pregnancy causes high glucose levels in maternal and fetus blood that can result in the fetus suffering, leading to complications such as early fetal death, congenital anomalies, macrosomia, and maybe long-term complications on the offspring (8, 9); in fact, according to the new concept of “metabolic memory”, the intrauterine hyperglycemia may act on the fetal hypothalamus and create a sort of “metabolic memory” that programs obesity and metabolic syndrome in the offspring during adulthood (10).

**Figure 1:**
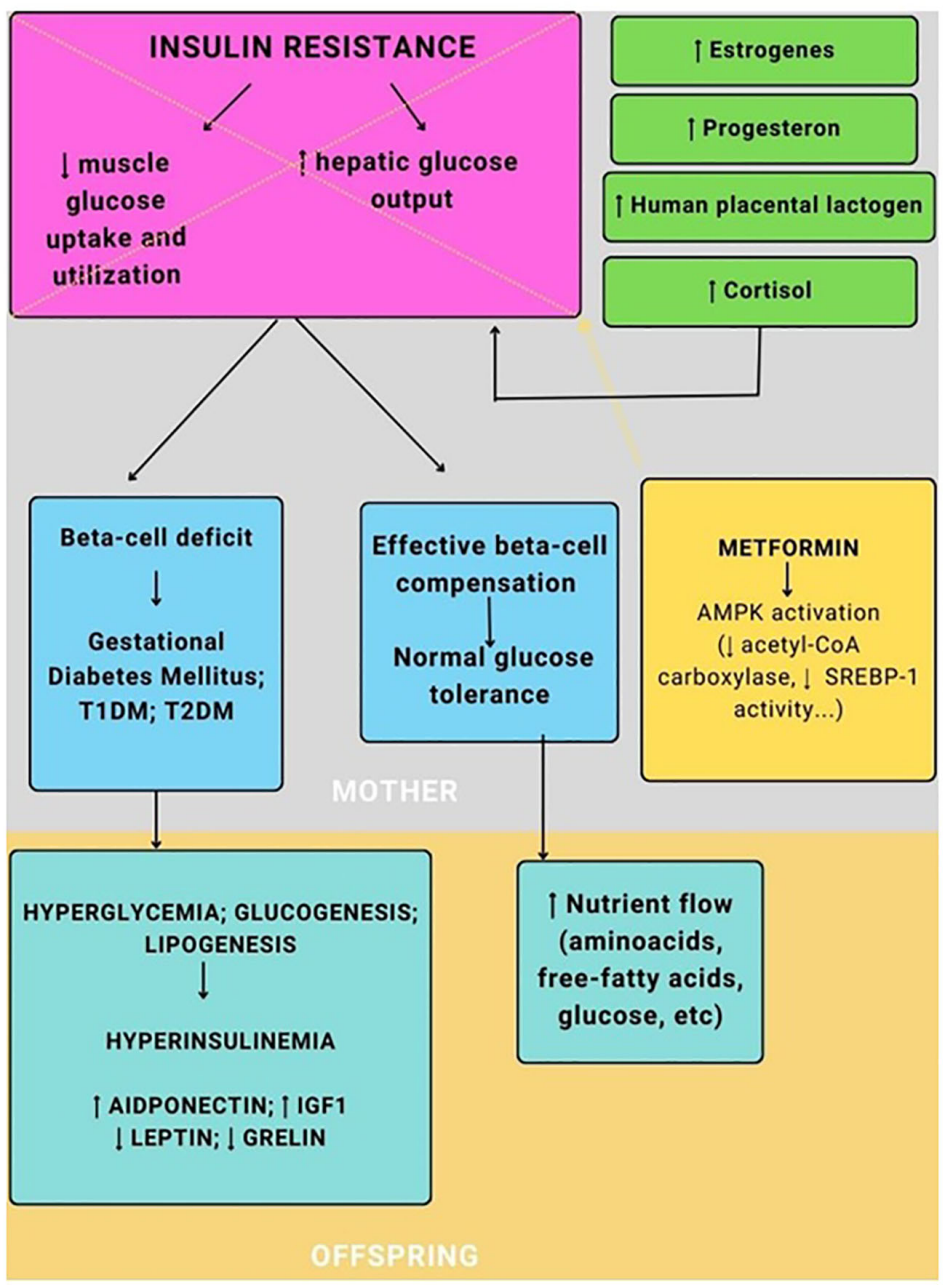
The pathogenesis of gestational diabetes and the role of metformin.

Therefore, a key role in the management of all these conditions, which, while implying a different pathological substrate, is linked by a common end effect (insulin resistance), could be played by metformin (**Figure 1**) (11).

In fact, metformin is a biguanide compound that has been shown to reduce hepatic glucose production, increase hepatic sensitivity to insulin, increase muscle glucose transport, and reduce hepatic steatosis by acting according to a complex picture in which a key role is played by AMP-activated protein kinase (AMPK) activation that activates a cascade of mechanisms including a reduction in acetyl-CoA carboxylase activity (12).

These mechanisms lead to a decrease in blood glucose level without a correlated elevated risk of hypoglycemia or weight gain (13).

Furthermore, because of its chemical and physical characteristics, metformin freely crosses the placenta, leading to a fetal level similar to maternal concentration (14). In addition, the safety of metformin in pregnancy is corroborated by a growing number of randomized clinical trials (15).

These characteristics make metformin a capital treatment for people with T2DM and an attractive drug for use in pregnancy (16). Not only that, but the characteristic properties of this small molecule could also allow us to use it in other conditions related to pregnancy such as lactation and fertilization.

Therefore, the aim of this literature review is to analyze the main available evidence on the use of metformin during, throughout, and beyond pregnancy. We included RCT studies and studies with an adequate sample size (at least 90 subjects, except for studies of particular relevance to our opinion).

## Metformin and gestational diabetes

One of the first studies to compare safety and efficacy of metformin vs. insulin in 63 women with gestational diabetes and similar baseline characteristics was published in 2007 by Moore et al. (17). Preliminary data showed no statistically significant difference in the rate of cesarean delivery (*p* = 0.102) and of neonatal characteristics at birth [birth weight, neonatal hypoglycemia, respiratory distress syndrome, hyperbilirubinemia, Apgar score at 5 min, and neonatal intensive care unit (NICU) admission (*p* = 0.144–0.373)].

After that, one of the most important studies on metformin in GDM was published in the *New England Journal of Medicine* in 2008 (18). The Metformin versus Insulin for the treatment of Gestational diabetes (MiG) study was an Australian off-label randomized trial including 751 women with GDM at 20 to 33 weeks of gestation to open treatment with metformin (373/751 patients) or insulin (378/751 patients). Inclusion criteria were 18–45 years of age, diagnosis of GDM with a single fetus between 20 and 33 weeks of gestation, and, after lifestyle intervention consisting of advice about diet and exercise, had more than one capillary blood glucose measurement >97.2 mg/dl after an overnight fast or more than one 2-h postprandial blood glucose measurement >120.6 mg/dl. The primary outcome was a composite of neonatal hypoglycemia, respiratory distress, need for phototherapy, birth trauma, 5-min Apgar score <7, or prematurity. Secondary outcomes included neonatal anthropometric measurements, maternal glycemic control, maternal hypertensive complications, postpartum glucose tolerance, and acceptability of treatment. The trial was designed to rule out a 33% increase (from 30% to 40%) in this composite outcome in infants of women treated with metformin as compared with those treated with insulin. Note that 168 women in the metformin arm (46%) required supplemental insulin. Those requiring insulin supplement had a higher body mass index (BMI) (*p* = 0.01), higher enrollment fasting glucose (*p* < 0.001), higher hemoglobin A1c (Hba1c) (*p* < 0.001), more frequent history of miscarriages (*p* < 0.001), less frequent nulliparous women (*p* = 0.003), and a higher representation of Polynesian ethnicity (*p* < 0.001).

The women clearly preferred metformin to insulin treatment and there was no difference in the composite primary outcomes (*p* = 0.95), and even in the single outcomes included in the composite, severe hypoglycemia was less common in the metformin group (*p* = 0.008), but preterm birth was more common in the metformin group (*p* = 0.04). Statistical significance in the secondary outcomes was found in the gestational age at birth (38.3 weeks in the metformin group vs. 38.5 weeks in the insulin group) and in the overall mean maternal 2-h postprandial glucose levels that were slightly lower in the metformin group (111.6 vs. 115.2 mg/dl); furthermore, women in the metformin group had greater weight loss between the time of enrollment and the postpartum visit (*p* = 0.006) and less weight gain between the time of enrollment and 36 weeks of gestation than did women in the insulin group (*p* < 0.001).

This important study, even with the limits of a wide enrollment range (20–33 weeks), a 2-year follow up that does not take into account lifestyle and feeding habits, and the fact that 46% of the women in metformin treatment required supplemental insulin, clearly shows that metformin is safe during pregnancy and is not associated with increased perinatal complications as compared with insulin.

Then, an open-label prospective randomized controlled trial (RCT) was published in 2010 involving 100 women with singleton pregnancies between 12 and 34 weeks of gestation with GDM in a secondary- and tertiary-level hospital in Finland who did not attain euglycemia with diet (<95 mg/dl fasting glucose and <120 mg/dl 2 h after meals) (19). They were randomized to therapy with insulin (*n* = 50) or oral metformin (*n* = 50).

The primary outcome was the incidence of macrosomia, defined as a birth weight >4,000 g, or large for gestational age (LGA). Neonatal complications, such as admission to NICU, hyperbilirubinemia treated with phototherapy, birth injuries (clavicular fracture or brachial nerve injury), and neonatal hypoglycemia requiring intravenous glucose treatment, were the secondary outcomes. The results show no statistically significant differences in the incidence of LGA (*p* = 0.97), mean birth weight, mean cord artery pH, or neonatal morbidity between the insulin and metformin groups. Fifteen out of 47 (31.9%) women randomized to metformin therapy needed supplemental insulin. The women needing supplemental insulin had higher mean BMIs (35.7 ± 7.2 vs. 29.6 ± 5.3 kg/m^2^, *p* = 0.002), had higher fasting capillary glucose concentrations (*p* = 0.001), and needed pharmacological treatment at earlier gestational age than women who were normoglycemic with metformin (26 ± 5.9 versus 31 ± 3.1 weeks), and their infant had higher birth weight (3,919 ± 400 versus 3,615 ± 417 g, *p* = 0.022).

In 2011, an observational study of all women with GDM who delivered after 20 weeks’ gestation at National Women’s Health from January 2007 to December 2009 was published (20). Since June 2007, women requiring glucose-lowering therapy could choose either metformin or insulin therapy, except for women with a fetal abdominal circumference < 10th percentile, who were not eligible for metformin. The study prospectively analyzed results from 1,269 women with GDM; 371 women were treated with diet, 399 women were treated with insulin, and 465 were treated with metformin (249 metformin alone and 216 metformin and insulin). Compared with those in the diet group, women taking metformin and/or insulin had higher fasting glucose at diagnosis (*p* < 0.001) and higher BMIs (*p* < 0.001). Women under insulin treatment had higher rates of cesarean section (CS) (45.6% insulin, 37% metformin, 34% diet, *p* = 0.02) than women under metformin or diet. Women under insulin treatment also had higher rates of preterm births (19.2% insulin, 12.5% metformin, 12.1% diet, *p* = 0.005), neonatal intravenous dextrose use (11.1% insulin, 5.1% metformin, 7.4% diet, *p* = 0.004), customized LGA infants (18.5% insulin, 12.5% metformin, 12.4% diet, *p* = 0.02), and NICU admissions (18.7% insulin, 12.7% metformin, 14.0% diet, *p* = 0.04).

An interesting finding is that, if we compare the patients on metformin plus insulin and the patients only on insulin, the group on metformin plus insulin had higher percentage of women with BMI > 30 (62.8 vs. 45.3%), a higher fast plasma glucose (5.7 vs. 5.4 mmol/L) on oral glucose tolerance test (OGTT), a higher percentage of CS (45.6% vs. 38%), and a higher percentage of preterm births (19.2 vs. 12.8%).

Niromanesh et al. in 2012 compared the efficacy of metformin and insulin in women with GDM (metformin *n* = 80, insulin *n* = 80), singleton pregnancy, and gestational age between 20 and 34 weeks, who did not achieve glycemic control and comparable maternal characteristics (21). The primary outcomes were maternal glycemic control and birth weight, while the secondary outcomes were neonatal and obstetric complications. Child born to women included in the metformin group had a lower rate of birth weight centile >90 than the insulin group (RR 0.5, 95% CI 0.3–0.9, *p* = 0.012) and maternal weight gain was reduced in the metformin group (*p* < 0.001), with comparable neonatal and obstetric complications (*p* > 0.05). Supplemental insulin was needed by 14% of women taking metformin in order to achieve glycemic control.

Metformin was, thus, indicated as an effective and safe alternative to insulin in women with GDM.

In 2012, a study was published to compare metformin with insulin as treatment of GDM and, furthermore, to characterize metformin-treated patients needing additional insulin to achieve prespecified glucose targets (99 mg/dl fasting glucose and 140 mg/dl 1 h after meal) (22).

It was a single-center randomized controlled study with a non-inferiority design comparing metformin and insulin in the treatment of 217 GDM patients. The primary outcome variable was the birth weight. No significant differences were found in the primary outcome. In the metformin group, 20.9% of the patients needed additional insulin. Factors predicting the need of additional insulin in metformin-treated patients were older age (*p* = 0.04), earlier gestational weeks at randomization (*p* = 0.004), earlier gestational week at OGTT (*p* = 0.01), higher Hba1C at randomization (5.6% vs. 5.44%, *p* = 0.01), and higher fructosamine at randomization (218.4 vs. 207.1 μmol/L, *p* < 0.001). Mothers with fructosamine concentration above the median before starting medication had a 4.6-fold (*p* = 0.006) higher probability for additional insulin than mothers having fructosamine below the median; the respective risk ratio for HbA1c between the patients having HbA1c above and below the median value was not significant (*p* = 0.09), leading us to hypothesize that fructosamine could be more useful than HbA1c in predicting the need of additional insulin.

A 2013 randomized trial of metformin vs. insulin in the management of GDM (23) including 97 pregnancy patients with GDM assigned to receive insulin (*n* = 47) or metformin (*n* = 47) showed lower weight gain (*p* = 0.002) and, moreover, a lower incidence of neonatal hypoglycemia (*p* = 0.032) in the metformin arm even if 26% of the metformin arm required an addition of insulin to their therapy.

It also showed that the probability of no response to metformin monotherapy was linked to earlier gestational age at diagnosis (*p* = 0.032) and mean pretreatment glucose level (*p* = 0.046).

A meta-analysis of five RCTs (some of them described above) was published in 2013 (24). It included 1,270 participants. Analysis of baseline characteristics showed that women requiring additional insulin had significantly higher fasting glycemic concentrations in OGTT (*p* = 0.0006).

The pooled results of main outcomes revealed that in the metformin group, there was a lower average weight gain after enrollment (*p* = 0.003), lower average gestational age at delivery (p = 0.02), higher incidence of preterm birth (*p* = 0.01), and lower incidence of pregnancy-induced hypertension (*p* = 0.02).

The limitations of this meta-analysis are linked not only to the high variability between the studies in the incidence of requiring additional insulin (especially high in the study of Rowan (18), 46.3%) but also to the high variability in metformin dose, the criteria of GDM’s diagnosis, and the glycemic targets.

Further confirmation on safety and efficacy of metformin treatment in pregnant women with GDM comes from a systematic review and meta-analysis of 24 studies published in 2021 (25). There were both maternal and neonatal outcomes. The neonatal outcomes comprehend birth weight, LGA, neonatal hypoglycemia, small for gestational age (SGA), macrosomia, NICU, Apgar score (<7) at 5 min, hyperbilirubinemia, respiratory distress syndrome (RDS), congenital anomalies, and umbilical cord pH. Maternal outcomes included gestational age at delivery, premature delivery, preeclampsia, pregnancy-induced hypertension (PIH), CS, maternal weight gain, and maternal glycemic control. Metformin was linked to lower risk of pregnancy-induced hypertension (*p* = 0.03), LGA babies (*p* = 0.04), macrosomia (*p* = 0.01), neonatal hypoglycemia (*p* = 0.001), and NICU admission (*p* = 0.01).

Another prospective trial published in 2021 (26) by a Spanish group evaluated metformin vs. insulin in 200 women with GDM (*n* = 100 in the metformin arm and *n* = 100 in the insulin arm). It was a multicenter, open-label, parallel-arm, randomized clinical trial enrolling women with singleton pregnancy, aged 18–45 years, with a gestational age between 14 and 35 weeks, and with GDM who needed pharmacologic treatment. The main outcomes of this study were glycemic control (mean glycemia and hypoglycemic events) and maternal and neonatal complications (hypertensive disorders of pregnancy, induced or spontaneous labor, preterm birth, fetal growth, neonatal care unit admission, respiratory distress syndrome, neonatal hypoglycemia, or jaundice requiring phototherapy). Metformin was started at 425 to 850 mg/day once or twice daily, and increased if necessary up to 2,550 mg/day. The insulin group was treated with detemir (0.2 UI/kg) plus, when necessary, aspart (0.1 UI/kg/meal).

This study confirmed that metformin was linked to less maternal weight gain (*p* = 0.011) and that there were no significant differences in birth weight, SGA, or LGA rates. The results show, differing from the MiG trial, that metformin was associated with lower postprandial glycemia and also reduced the rate of delivery by CS compared to those treated with insulin (*p* = 0.001).

A slightly different role of metformin was explored in the double-blind, multicenter, randomized trial of Valdes et al. published in 2019 in the *Journal of Obstetrics and Gynecology Research* (27). The aim of this study was to evaluate the role of metformin in the prevention of GDM in pregnant women with pregestational insulin resistance. They recruited 140 patients randomly assigned to take metformin (*n* = 68) or placebo (*n* = 73). The results show that patients in the metformin group did not have a decrease in the incidence of GDM as compared to placebo (37.5% vs. 25.4%, respectively; *p* = 0.2), but they experienced a higher incidence of drug intolerance (*p* = 0.02).

Mean results of the most important studies mentioned above are listed in **Table 1**.

**Table 1:** Metformin for the treatment of women with GDM.

Trial	*N*	Gestational week at inclusion	Metformin dose (mg)	Comparator	CS	PE	GWG	BW	LGA	SGA	Preterm births	Other neonatal outcomes
Moore 2007 (17)	63	24–30	1,000–2,000	Insulin	=	/	/	=	/	/	/	=
MiG 2008 (18)	751	20–33	2,500 ± insulin	Insulin	/	=	<	=	=	=	>	< Neonatal hypoglycemia
Ijäs 2010 (19)	100	12–34	2,250 ± insulin	Insulin	>	=	=	=	=	/	=	=
Goh 2011 (20)	1,269	/	2,500 ± insulin	Insulin/dayt	<	=	/	=	<	=	<	< NICU ≥ 2 days
Niromanesh 2012 (21)	160	20–34	1,000–2,500 ± insulin	Insulin	=	=	<	<	<	=	=	=
Tertti 2012 (22)	217	22–34	1,500 ± insulin	Insulin	=	=	=	=	=	/	=	=
Spaulonci 2013 (23)	97	/	1,700–2,550	Insulin	=	=	<	=	=	=	=	< Neonatal hypoglycemia
Picón-César 2021 (24)	200	14–35	425–2,550	Insulin	<	/	<	=	=	=	=	=

N, number; CS, cesarean section; PE, preeclampsia; GWG, gestational weight gain; BW, birth weight; LGA, large for gestational age; SGA, small for gestational age. < increased incidence. > decreased incidence. = unchanged incidence. / variable not analyzed.

These studies show that, in GDM, metformin is safe and effective; it is linked to less weight gain and a lower risk of neonatal hypoglycemia compared to insulin treatment.

The limitation of these lines of evidence is linked to a scarcity of randomized clinical trials, most with a small number of patients included, and no clinical studies designed with the purpose of evaluating the efficacy and safety of the metformin and insulin combination treatment, and that data from metformin and insulin combined use are derived from metformin vs. insulin comparison studies or retrospective data (non-randomized controlled trials).

The positive effects of metformin are linked to the increase in the hepatic and peripheral uptake of glucose, the reduction in hepatic output of glucose, and the increase in insulin sensitivity. Also not to be underestimated is the fact that metformin, in addition to being safe and effective in this subgroup, is a low-cost molecule, with a low risk of hypoglycemia, and does not require educational programs or intensive control of glycemia.

Concerns about the use of metformin in GDM are linked to the transplacental passage of this molecule and to the high concentration in the umbilical artery and vein.

The disadvantages of metformin are moreover linked to collateral effects like nausea and/or vomiting, diarrhea, and the uncertainty on fetus’ long-term effects.

## Metformin treatment in pregnant women with T2DM

The most important study to evaluate the safety and efficacy of metformin in pregnant women affected by T2DM is the MiTy trial, a randomized double-blind multicenter international placebo-controlled study involving 502 women with T2DM under insulin therapy between 18 and 45 years old and 6 to 22 weeks plus 6 days of pregnancy randomly assigned to take metformin 1 g twice daily (*n* = 253) or placebo one capsule twice daily (*n* = 249) published in 2020 (28). The primary outcome was a composite of fetal and neonatal outcomes (pregnancy loss, preterm birth, birth injury, moderate or severe respiratory distress syndrome, neonatal hypoglycemia, and NICU admission lasting >24 h). Secondary outcomes included maternal glycemic control, maternal hypertensive disorders, CS, gestational weight gain and insulin dose, LGA, extreme LGA, SGA, cord blood C-peptide, neonatal adiposity outcomes, gestational age at birth, and length of infant hospital stay.

They found no significant difference in the primary composite neonatal outcome between the two groups (40% vs. 40%, *p* = 0.86) but compared with women in the placebo group, metformin-treated women had lower levels of Hba1c, gained less weight, and had a lower incidence of CS; metformin-exposed infants had lower birth weight (*p* = 0.0016), a lower incidence of extreme LGA (22% vs. 27%, *p* = 0.041), and a higher incidence of SGA (13% vs. 7%, *p* = 0.026), and infants had slightly higher incidence of neonatal jaundice (23% vs. 16%, *p* = 0.06) (**Table 2**).

**Table 2:** Metformin for the treatment of pregnant women with T2DM.

Trial	*N*	Gestational week at inclusion	Metformin dose (mg)	Comparator	CS	PE	GWG	BW	LGA	SGA	Preterm births	Other neonatal outcomes
Mity trial (28)	502	6–22	2,000 + insulin	Placebo + insulin	<	=	<	<	=	>	=	=

N, number; CS, cesarean section; PE, preeclampsia; GWG, gestational weight gain; BW, birth weight; LGA, large for gestational age; SGA, small for gestational age. < increased incidence. > decreased incidence. = unchanged incidence.

## Metformin treatment in pregnant women with obesity

In 2015 the EMPOWaR was published, a randomized double-blind placebo controlled study involving 449 pregnant women (aged ≥16 years) between 12 and 16 weeks’ gestation who had a BMI of 30 kg/m^2^ or more and normal glucose tolerance in 15 National Health Service hospitals in the UK (29). Women were randomly assigned (1:1) to metformin (maximum 2,500 mg/day) or placebo. Demographic characteristics, comorbidities, and anthropometric parameters overlap between the two groups. Primary outcome was birth weight percentile. Secondary outcomes were insulin resistance at 36 weeks EG, maternal fasting glucose and insulin at 2 h of glucose load at 36 weeks CE, maternal and neonatal anthropometric parameters, maternal inflammatory markers, and incidence of IUGR. There were no significant differences in the primary outcome or in the secondary outcomes, which shows that metformin had no significant effect on birth weight percentile in obese pregnant women.

One of the most important studies to explore the role of metformin on obese pregnant patients was the GRoW trial, a randomized double-blind placebo-controlled study on 524 women with a BMI >25 kg/m^2^ of 10–20 weeks of gestation; 256 women were randomly assigned to take metformin 2,000 mg/day, and 258 women took placebo (30). This study shows no differences in GDM incidence or in the incidence of other maternal complications like hypertension or preeclampsia except for a lower weight gain per week in the metformin group (*p* = 0.006). No differences were found either in the incidence of perinatal adverse outcomes including macrosomia or LGA.

In 2020, a randomized clinical trial involving 357 obese (BMI >30 kg/m^2^) pregnant women without diabetes was published; 186 women were assigned to take placebo and 171 were assigned to take metformin (31). The main outcomes were absolute risk reduction and the number of women who needed treatment for CS and LGA. There were no differences in patients’ baseline characteristics except for marital status.

The incidence rate of CS in the metformin group was 39.8% vs. 62.9% in the control group (*p* < 0.01). No differences were found in the LGA prevention. Between the maternal–fetal outcomes assessed in the secondary analysis (GDM, preeclampsia, prematurity, newborn weight, SGA, Apgar 1st and 5th min, and NICU), only the incidence of preeclampsia seems to be reduced in the metformin group (*p* < 0.01).

In 2020, another study was published on metformin to analyze its role in the lipid profile, BMI, and weight gain of pregnant women with obesity (32). This study was a randomized clinical trial involving 436 obese pregnant women randomly assigned to low-dose metformin (*n* = 206) or control (*n* = 218). The inclusion criteria were pregnant women with obesity ≥18 years, single pregnancy, negative screening for GDM in early pregnancy, and gestational age <20 weeks.

There was no difference in lipid profile, BMI, and weight gain values between groups during the 1st, 2nd, and 3rd evaluation. A significant difference was observed only in the BMI, high-density-lipoprotein (HDL), and triglycerides (TG) values from the 1st to 3rd evaluation.

We can conclude that in obese women, even if metformin seems to be potentially useful in the inflammatory response modulation and in the severe obesity to reduce the risk of preeclampsia, there is no evidence that, in this subgroup of patients, metformin has a role in fetal growth; it does not reduce the risk of GDM, it does not improve maternal lipidic profile, and benefits have only been observed in some of the outcomes addressed (**Table 3**).

**Table 3:** Metformin for the treatment of pregnant women with obesity.

Trial	*N*	Gestational week at inclusion	Metformin dose (mg)	Comparator	CS	PE	GWG	Maternal lipid assessment	BW	LGA	SGA	Preterm births	Other maternal/neonatal outcomes
EMPOWaR 2015 (29)	449	12–16	2,500	Placebo	=	=	=	=	=	/	=	=	< Admission to neonatal unit
GRoW 2019 (30)	524	10–20	2,000	Placebo	<	=	<	/	=	=	=	=	=
Nascimento 2020 (31)	357	<20 weeks	1,000	Placebo	<	<	/	/	/	=	/	=	=
Dienstmann 2020 (32)	436	<20 weeks	1,000	Placebo	/	/	=	=	/	/	/	/	=

N, number; CS, cesarean section; PE, preeclampsia; GWG, gestational weight gain; BW, birth weight; LGA, large for gestational age; SGA, small for gestational age. > decreased incidence. = unchanged incidence. / variable not analyzed.

## Metformin treatment in pregnant women with PCOS

A prospective, randomized, double-blind, placebo-controlled pilot study to investigate a possible effect of metformin on androgen levels in pregnant women with PCOS was published in 2004 (33). Forty pregnant women with PCOS were randomly assigned to receive diet and lifestyle counseling plus metformin 850 mg/day twice daily or plus placebo.

Primary outcome measures were dehydroepiandrosterone sulfate (DHEAS), androstenedione, testosterone, SHBG, and free testosterone index (FTI). Secondary outcome measures were pregnancy outcome and pregnancy complications. Metformin had no effect on maternal androgen levels in pregnant women with PCOS. Moreover, while none of the 18 women in the metformin group experienced a severe pregnancy or post-partum complication, 7 of the 22 (32%) women in the placebo group experienced severe complications (*p* = 0.01), showing promising results for overall pregnancy complications.

The PregMet study was published in 2010 (34). It was a prospective, randomized, double-blind, multicenter trial comparing metformin 2,000 mg daily with placebo in 257 women with a history of PCOS aged 18–42 years and enrolled in the first semester of pregnancy. Primary outcomes were the prevalence of preeclampsia, preterm delivery, GDM, and a composite of these three outcomes. Secondary outcomes included weight, blood pressure, heart rate, and mode and length of delivery.

The results show no differences between the groups in the prevalence of preeclampsia, preterm delivery, GDM, or the composite of these three pregnancy complications, contradicting the previous pilot study mentioned above. Between secondary outcomes, they only found a significant difference in weight gain with a lower increase in the metformin group.

Pooled data from these two studies showed a significant reduction in the combined endpoint of late miscarriage and preterm birth in favor of metformin (35).

One of the biggest studies involving pregnant women with PCOS is the PregMet2, a randomized, double-blind, placebo-controlled study involving 487 singleton pregnant PCOS women aged 18–45 years randomly assigned to metformin (*n* = 244) or placebo (*n* = 243) published in 2019 (36). The primary outcome of this intention-to-treat analysis was incidence of late miscarriage (EG 13 -22 + 6) and preterm birth (EG 23 -36 + 6); the secondary outcomes were incidence of GDM, preeclampsia, pregnancy-induced hypertension, and admission of the neonate to the NICU, and tertiary outcome was weight gain in pregnancy, from inclusion until week 36. Women in need of assisted reproductive technology (15%–20%) were equally distributed between the treatment groups. The different phenotypes of PCOS were equally distributed between the treatment groups. Metformin’s starting dose was 500 mg twice daily during the first week of treatment, increased to 1,000 mg twice daily from week 2 until delivery. Treatment was started in the first trimester as soon as possible, and at the latest 7 days after, the inclusion visit. If necessary because of side effects, doses were adjusted to an acceptable level. All women received diet and lifestyle advice according to national guidelines. The results of PregMet2 showed a non-significant reduction in the incidence of late miscarriage or preterm delivery (primary outcome). No substantial between-group differences were found in maternal and offspring adverse events (secondary outcomes). Women in the metformin group gained less weight from inclusion to gestational week 36 compared with those in the placebo group (*p* < 0.001).

An interesting study was made on a subgroup of PregMet2 enrolled women (*n* = 73) who agreed to provide serum sample at three time points in pregnancy (gestational weeks 19, 28, and 32) and once in postpartum, (either 2, 4, or 8 weeks after delivery) (37). The study showed an increase of 32% in metformin concentration already during the first 2 weeks postpartum, probably linked to the resolution of the pregnancy hemodilution. These results may impact both the therapeutic efficacy during pregnancy and the risk of adverse drug reactions that could be higher postpartum.

The results of PregMet2 partially contradict a previous meta-analysis based on 13 studies including 5 RCTs and 8 cohort studies involving 1,606 pregnant women with PCOS published in 2016 (38).

The primary outcomes of this meta-analysis included early pregnancy loss, preterm delivery, term delivery, and GDM; secondary outcomes included pregnancy-induced hypertension (PIH), intrauterine growth restriction (IUGR), fetal malformation, vaginal delivery (VD), CS, and metformin’s side effects, such as nausea or gastrointestinal discomfort. Taking metformin seems to reduce the risk of miscarriage, preterm delivery, complications like GDM, hypertensive disorders, and CS. However, the positive effect on GDM incidence was not confirmed in sub-analysis including only the randomized trials.

Mean results of the three most important studies mentioned above are listed in **Table 4**.

**Table 4:** Metformin for the treatment of pregnant women with PCOS.

Trial	*N*	Gestational week at inclusion	Metformin dose (mg)	Comparator	CS	PE	GWG	BW	LGA	SGA	Preterm births	GDM
Pilot study 2004 (33)	40	5–12	1,700 mg	Placebo	/	/	/	=	/	/	/	/
PregMet 2010 (34)	257	5–12	2,000	Placebo	=	=	<	=	/	/	=	=
PregMet 2 2019 (36)	487	6–12	2,000	Placebo	=	=	<	=	/	/	=	=

N, number; CS, cesarean section; PE, preeclampsia; GWG, gestational weight gain; BW, birth weight; LGA, large for gestational age; GDM, gestational diabetes; SGA, small for gestational age. < increased incidence. > decreased incidence. = unchanged incidence. / variable not analyzed.

## Metformin and fertilization

PCOS is among the most common endocrinopathies associated with reproductive and metabolic disorders and affects 9% to 18% of women (37). According to the World Health Organization, it belongs to group II of ovulation disorders and accounts for 80% of women with anovulatory syndrome (39).

The Rotterdam criteria (2003) for the diagnosis of PCOS require that women must meet two of the following items: oligo-ovulation or anovulation, clinical and/or biochemical signs of hyperandrogenism, and polycystic ovaries (40).

Among the most frequent clinical manifestations of PCOS, there are irregular periods, infertility, hirsutism, acne, obesity, inappropriate gonadotropin secretion (i.e., elevated levels of circulating luteinizing hormone), pregnancy complication, cardiovascular disease, and metabolic features including especially insulin resistance with compensatory hyperinsulinemia (40, 41).

The reduction of insulin resistance has been proven to improve ovulation and fertility in women with PCOS, and this led to many studies regarding the possible role of insulin-sensitizing agents, particularly metformin, in the treatment of PCOS (41, 42). Metformin reduces hyperinsulinemia and suppresses the excessive ovarian production of androgens (43). It is suggested that, as a consequence, metformin could improve assisted reproductive technique (ART) outcomes, such as ovarian hyperstimulation syndrome (OHSS), pregnancy, and live birth rates.

Despite the multitude of RCTs conducted, high-quality RCTs designed to answer the specific question of the comparative efficacy of metformin in patients with PCOS and with or without obesity are still lacking (39). Some limited studies have found that BMI may affect the efficacy of metformin (44, 45).

Wu et al. conducted a study that aimed to systematically review the literature and performed a meta-analysis in 2020 to clarify whether metformin is associated with improved outcomes in women with PCOS undergoing *in vitro* fertilization or intracytoplasmic sperm injection and embryo transfer (IVF/ICSI-ET) cycles (41). It included 12 studies involving 1,123 women with PCOS undergoing IVF/ICSI-ET, and its outcomes were OHSS rate, clinical pregnancy rate, live birth rate, and miscarriage rate.

Women in the metformin group had lower odds of OHSS than women in the control group (OR 0.43; 95% CI 0.24–0.78); in particular, women in the subgroup with BMI > 26 had lower rates of OHSS if randomized with metformin (OR 0.25, 95% CI 0.12–0.51). No differences were observed in OHSS rate in the subgroup with BMI < 26.

Metformin was not associated with the clinical pregnancy rate (OR 1.24, 95% CI 0.82–1.86). Dividing two groups by BMI, there was a significant difference in clinical pregnancy rate (OR 1.7, 95% CI 1.12–2.60) in the subgroup with BMI > 26 treated with metformin.

There was no evidence of a difference in live birth rate between the metformin and control groups (OR 1.23 95% CI 0.74–2.04), even dividing the two subgroups by BMI, or in miscarriage rate (OR 0.58 95% CI 0.24–1.39).

Tso et al. conducted a systematic review published in the Cochrane Database to determine the effectiveness and safety of metformin as a co-treatment during IVF or ICSI in achieving pregnancy or live birth in women with PCOS (43). It included 13 studies for a total population of 1,132 women of reproductive age with anovulation due to PCOS with or without coexisting infertility factors. They stratified the analysis by type of ovarian stimulation protocol used [long gonadotropin-releasing hormone agonist (GnRH agonist) or short gonadotropin-releasing hormone antagonist (GnRH antagonist)] to determine whether the type of stimulation used influenced the outcomes.

The review showed uncertainty of the effect of metformin on live birth rate when compared to placebo/no treatment (RR 1.30, 95% CI 0.94–1.79) for the GnRH-agonist group, while it may reduce live birth rate for the GnRH-antagonist group.

Metformin could reduce the incidence of OHSS (RR 0.46, 95% CI 0.29–0.72), while regarding the clinical pregnancy rate, it demonstrated an increase in women of the GnRH-agonist group (RR 1.32, 95% CI 1.08–1.63), and uncertainty for the GnRH–antagonist group.

Metformin may also result in an increase in side effects (mainly gastrointestinal) compared with placebo/no treatment (RR 3.35, 95% CI 2.34–4.79).

The overall quality of evidence ranged from very low to low.

In conclusion, this review found no conclusive evidence that metformin improves live birth rates; in a long GnRH-antagonist protocol, it is uncertain whether metformin improves live birth rates, but it may increase the clinical pregnancy rate; in a short GnRH-antagonist protocol, metformin could reduce live birth rates, with uncertainty on clinical pregnancy rate. Metformin could also reduce the incidence of OHSS in the long GnRH-agonist protocol but not in the GnRH-antagonist ovarian stimulation protocol (**Table 5**).

**Table 5:** Metformin to improve fertilization.

Study	*N*	Comparator	OHSS		Clinical pregnancy rate		Live birth rate		Miscarriage
Wu et al. 2020 (41)	12 studies 1,123 women	Metformin vs. control	BMI < 26	BMI > 26	BMI < 26	BMI > 26	=		=
			=	<	/	>			
Tso et al. 2020 (43)	13 studies 1,132 women	Metformin vs. placebo/no treatment	<		GNRH-antagonist	GNRH-agonist	GNRH-antagonist	GNRH-agonist	/
					U	>	<	U	

N, number; BMI, body mass index; OHSS, ovarian hyperstimulation syndrome; GNRH, gonadotropin releasing hormone; U, uncertain.

Another meta-analysis summarized 47 studies and concluded that metformin could lower triglyceride levels in patients with PCOS who did not have diabetes, possibly through improving oxidative stress status (46).

One guideline (42) pointed out that stopping metformin treatment at the initiation of gestation did not influence the live birth rate; however, the already mentioned study of PregMet2 (36) stated that metformin could reduce the incidence of late miscarriage and preterm birth when the treatment is prolonged to the late first trimester to delivery.

## Metformin and breastfeeding

Maternal obesity is consistently associated with delayed lactogenesis (47, 48). Recent studies revealed the different insulin sensitivity of the mammary gland during pregnancy and lactation (49).

Nommsen et al. designed a metformin-versus-placebo randomized clinical trial involving 15 women with insulin resistance and low milk production despite regular breast emptying; their hypothesis was that an intervention targeting insulin action could improve milk production (49). Metformin is considered compatible with lactation, with milk concentrations from 0.1 to 0.4 mg/L and undetectable or very low detection of metformin (<0.08 mg/L) in the serum of breastfed infants. Women took metformin at a dosage of 750 mg/day from day 1 to 7, 1,500 mg/day from day 8 to 14, and 2,000 mg/day from day 14 to 28. They measured breast milk production by having participants weigh their infants on a specialized scale immediately before and after feeding on each breast over 24 h for 14–28 days. They found that maximum milk production improved from baseline in 60% of the participants who were assigned metformin and in 20% of the placebo group. Median change in milk production was 68 ml greater in participants assigned metformin as compared to placebo participants, with a non-statistically significant difference. *Post-hoc* results led to the conclusion that an intervention aimed at improving insulin sensitivity could improve milk production (median peak change in milk output +22 in metformin completers *n* = 8, versus −58 ml/24 h placebo + non-completers, *n* = 7), even though absolute milk output remained very low even in the participants who completed the metformin course.

## Metformin and offspring

It is known that offspring of women with diabetes have an increased fat mass at birth but not an increase in fat-free mass (50), an explanation could be the continued exposure to nutrient excess in the uterus that may cause an overload of the subcutaneous fat stores and, thus, the development of leptin and insulin resistance and a deposit of excess nutrients as ectopic fat (51). Reduced insulin sensitivity has been demonstrated in cord blood of infants exposed to maternal hyperglycemia (52).

Moreover, large-scale epidemiological studies have highlighted how the offspring of obese women have an increased incidence of reduced cognitive performance (53), attention deficit hyperactivity disorder (ADHD) (54), psychiatric disorders (55), cerebral paralysis (56), and autism spectrum disorders (57).

Women with insulin resistance then need to achieve strict glycemic control to avoid pregnancy complications resulting from hyperglycemia (58), and insulin is proven to reduce complications for both the mother and the fetus (5).

GDM not properly controlled with diet is commonly treated with insulin, although there are different guidelines: the American Diabetes association recommends using insulin as first-line treatment for GDM, the National Institute for Health and Care Excellence (NICE) proposes the use of metformin as first-line treatment, and the Society for Maternal Fetal Medicine considers metformin a reasonable and safe first-line pharmacologic alternative to insulin (57, 59, 60).

Metformin use during pregnancy has been studied mainly for PCOS and GDM, and the main worries come from the fact that it freely crosses the placenta and reaches a fetal level similar to maternal concentration (61). It is possible that metformin exposure in the uterus might lead to improved insulin action in the fetus, resulting in a metabolically healthier pattern of growth, but it remains extremely important to examine longer-term outcomes (24). A lack of long-term offspring follow-up data has led to caution about using metformin routinely in GDM.

The MiG-TOFU is a series of studies that, starting from the results of the MIG trial (17), tried to assess potential effects on growth of the children.

Rowan et al. led an offspring follow-up (TOFU) investigating the body composition at 2 years of age (62). In Auckland and Adelaide, women who had participated in the MiG trial were reviewed when their children were 2 years old and the children were assessed with anthropometrics, bioimpedance, and DEXA. There were no differences between groups in the baseline characteristics of mothers at the randomization of treatment, and there were no differences between groups in measurements at birth, maternal glucose control during pregnancy, and rates of breastfeeding at 6/8 weeks postpartum. Their first hypothesis was that metformin exposure in the uterus would be associated with less central fat and, then, less insulin resistance in the offspring. Body composition measurements at 2 years of age showed three significant differences: the upper arm circumference was larger in the metformin group, and subscapular skinfolds and biceps skinfolds were bigger, while there were no differences in DEXA and bioimpedance measures. Their first hypothesis was not confirmed though, since they found no differences between the two groups in central fat measures, total fat mass, percentage body fat, or central to peripheral fat. Anyway, the larger skin folds may suggest that exposure to metformin has led to more fat being stored in subcutaneous sites, which may, in turn, mean that there is less ectopic or visceral fat in these children.

The importance of these findings lies in the fact that size and location of the fat cells are important predictors of insulin resistance and adverse metabolic consequences of obesity (51, 63, 64). They provide feedback about food intake and satiety, and in situations of excessive nutrient intake, the adipocytes become large and dysfunctional and excess fat is deposited in visceral adipocyte depots, which release fatty acids and inflammatory adipocytokines, associated with insulin resistance (64).

The same authors led another study, investigating body composition and metabolic outcomes at 7–9 years of age of the same children of the previous MiG TOFU (65). Its aim was to compare body composition and markers of insulin sensitivity between the groups treated with metformin and insulin.

The Adelaide subgroup was assessed at 7 years of age and all measures of body composition, adjusted for age, gender, and ethnicity, were similar in the two groups. In the Auckland subgroup, at 9 years of age, the metformin group was still larger on several measures, including weight, mid-upper arm circumference, waist circumference, and waist-to-height ratio. They also had a trend toward higher fat-free mass and fat mass. In conclusion, that study reported similar total and abdominal fat body percentage and metabolic measures in 7- to 9-year-old offspring of women randomized to metformin or insulin treatment during pregnancy, even if 9-year-old offspring of women randomized to metformin were larger than those whose mothers had been randomized to insulin.

Paavilainem et al. compared the lipid and glucose metabolism in 9-year-old offspring of mothers treated with metformin or insulin for GDM, beyond anthropometrics (66). It was the result of a longitudinal follow-up study of two previously published Finnish RCTs with a similar study design, including a total of 172 children, already mentioned above (19, 22).

Neonatal measures, such as birth weight, crown–heel length, ponderal index, and sex distribution, did not differ significantly between the two groups. Maternal baseline characteristics, pregnancy outcomes, and neonatal measures were also found to be similar in the two groups. At the follow-up evaluation, all the 9-year-old children were prepubertal. There were no significant differences between the metformin and insulin groups in terms of weight, height, BMI, proportion of overweight or obese children, waist circumference, waist-to-height ratio, and systolic or diastolic blood pressure. Data were consistent with existing literature.

Regarding the metabolic profile, the offspring on the metformin group were found to have a more favorable lipid profile. Their HDL cholesterol concentration was higher, whereas their low-density-lipoprotein (LDL) and apolipoprotein B concentrations were lower, although the significance in HDL increased concentration was reached only in boys. The glucose metabolism values (fasting glucose, fasting insulin, fasting C peptide, HbA1C, and OGTT) were similar.

Mean results of the three studies mentioned above are listed in **Table 6**.

**Table 6:** Impact of metformin treatment during pregnancy on offspring.

Trial	Comparator	Subgroups	Measures	Results
MIG-TOFU 2 years 2011 (62)	Metformin vs. insulin		Upper arm circumference	>
			Subscapular skinfolds	>
			Biceps skinfolds	>
			Central fat measures	=
			Total fat mass	=
			Percentage body fat	=
			Central to peripheral fat	=
MIG-TOFU 7–9 years 2017 (65)	Metformin vs. insulin	Adelaide group 7-year-olds	Body composition	=
		Auckland group 9-year-olds	Weight Mid-upper arm circumference Waist circumference Waist-to-height ratio Fat-free mass	>
Paavilainen 9 years 2022 (66)	Metformin vs. insulin		Weight Height BMI Overweight/obese % Waist circumference Waist-to-height ratio	=

< increased incidence. = unchanged incidence.

Exploring the cardiovascular profile in the offspring, Panagiotopoulou et al. designed a follow-up study, including 151 children from the Metformin (vs. Placebo) in Obese Pregnant Women trial (67) to assess whether prenatal exposure to metformin can improve the cardiovascular profile and body composition in the offspring of obese mothers (68). Consistently with other studies, they found no differences in weight, height, body mass index, skinfolds, and body fat distribution measurements. The rate of weight gain from birth to early childhood (children were about 4 years old at the time of the evaluation) was also comparable.

On the cardiovascular side, children in the metformin group had shorter isovolumetric relaxation time and smaller left atrial area and higher pulmonary vein peak systolic Doppler velocity value. Measures of cardiac systolic function were similar.

Regarding hemodynamic parameters and vascular phenotype, there was no significant difference in peripheral systolic blood pressure and diastolic blood pressure. After multivariable adjustments, children exposed to metformin had lower aortic pulse pressure and aortic systolic blood pressure, suggesting a reduced central blood pressure with an improvement in central hemodynamics and left ventricular diastolic indices. These results suggest a putative beneficial and protective effect of metformin to the cardiovascular system of the offspring.

The PedMet study (69), led by Hanem et al., was conducted with different results exploring the cardiometabolic risk factors in children during a follow up of the PregMet study, a randomized, placebo-controlled, double-blind study that investigated the role of metformin in women with PCOS during pregnancy in terms of reduction of pregnancy complications (34). They re-evaluated children after 5/10 years and concluded that children in metformin groups vs. placebo had a higher BMI *Z* score, a higher measure of abdominal adiposity, and a higher weight. There was no difference in height *Z* score, head circumference *Z* score, adiponectin, cholesterol, TG, HDL cholesterol, non-HDL cholesterol, alanine aminotransferase, glucose, HbA1c, insulin, c-peptide, Homeostasis Model Assessment 2-Insulin Resistance (HOMA2-IR), blood pressure, or heart rate.

As with the majority of the studies available in literature, all the aforementioned studies did not differentiate metformin use alone or in combination with insulin, in comparison with insulin or placebo.

Brand et al. instead designed a register-based cohort study, including more than 10,000 children with maternal exposure to metformin or insulin regardless of the indication (GDM, presentational T2DM, or PCOS), classified into three exposure groups: metformin, insulin, and combination treatment (15). As primary outcomes, they demonstrated that for obesity and hypoglycemia, the incidence was higher for the combination treatment; for hyperglycemia, there were no marked differences between the groups. Moreover, for motor–social development, no significant difference was observed. No events of hypertension or PCOS were observed in the metformin or combination treatment groups.

As secondary outcomes, exposure to metformin was associated with significantly lower mean birth weight, and compared with insulin, no differences were observed for the other secondary outcomes (LGA, SGA, preterm birth, neonatal mortality, neonatal hypoglycemia and hyperglycemia, and major congenital anomalies).

In the CogMet study, Greger et al. explored whether metformin (vs. placebo) exposure in the uterus had any effect on offspring cognitive function (70). The study was designed as a follow-up of two randomized, placebo-controlled studies [the pilot study (33) and the PregMet study (34)], and included 93 children with a mean age of 7.7 years. There was no difference between participants and nonparticipants regarding maternal baseline data, pregnancy outcomes, and neonatal data. All anthropometric measures, including Tanner stage development of the children at follow-up, were also comparable in the two groups.

The mean full-scale intelligence quotient (FIQ) in the metformin and placebo groups were similar and corresponded to the average FIQ score in the background population. There were no statistically significant differences on the subscales (verbal comprehension, working memory, perceptual organization, or processing speed), and the results did not change after adjustment for maternal/paternal educational level.

## Conclusion

### What do the results of these studies tell us?

Clinical and scientific evidence presented above support the safety and efficacy of metformin during pregnancy. In pregnant women with GDM and T2DM, metformin improves obstetric and perinatal outcomes, but there is no evidence that metformin prevents GDM in women with pregestational insulin resistance (27). In addition, no improvement in lipid profile and risk of GDM was demonstrated in pregnant women with PCOS or obesity (32). Metformin could have a role in reducing the risk of preeclampsia in pregnant women with severe obesity (31) and the risk of late miscarriages and preterm delivery in women with PCOS (38). In women with PCOS undergoing IVF/FIVET, taking metformin seems to be associated with a lower risk of OHSS (40).

Offspring of mothers exposed to metformin have no significant differences in long-term outcomes compared to those born to mothers exposed to insulin (63, 64, 68).

Maternal exposure to metformin and combination treatment of metformin and insulin was not associated with long-term increased risk of obesity, hypoglycemia, hyperglycemia, diabetes, or challenges in MSD compared with insulin. The analyses of adverse outcomes at birth showed significantly lower birth weight and significantly increased risk of SGA associated with exposure to metformin, compared with insulin; combination treatment was associated with increased risk of LGA, preterm birth, and hypoglycemia (15).

Metformin in pregnancy appears to be protective for metabolic risk in babies to mothers with GDM (66) and cardiovascular risk in babies born to obese mothers (68).

Metformin in pregnancy appears to increase metabolic risk in babies born to mothers with PCOS (69).

It would be of great interest to evaluate glycemic profiles with subcutaneous continuous monitoring devices and also to compare new long-acting formulations of insulin among them and with metformin (26).

Furthermore, there are not enough studies reporting long-term data nowadays, and whether the effect of metformin will continue until adulthood is an important point to explore.

### What do the guidelines tell us?

Despite the clinical and scientific lines of evidence listed above, the Italian standards for the treatment of diabetes mellitus 2018 declare that in all women with GDM or T2DM in whom the glycemic target is not achievable by diet alone, insulin therapy should be promptly instituted; oral antidiabetics and non-insulin injection therapy are currently not recommended in pregnancy; a possible introduction of metformin into the GDM therapy remains suspended pending reliable data on its long-term safety in the fetus and offspring (71).

The global guideline on pregnancy and diabetes published in 2017 instead declares that women with T2DM who are taking metformin during pregnancy need information about the potential advantages and disadvantages of these medications; for women with GDM not controlled by diet, insulin is the treatment of choice; however, metformin can be considered a safe and effective alternative (72).

The National Institute for Health and Clinical Excellence (NICE) and the Canadian Diabetes Association include metformin as an option for treatment of GDM, and NICE also includes metformin as an option for the treatment of T2DM in pregnancy, even if it is not licensed for these indications (61, 73).

### A clinical approach

An interesting review recently published proposed a clinical targeted approach in the use of metformin in pregnant women (74).

In obese pregnant women, even if on a small evidence base, metformin could have a role in very obese women (BMI >35 kg/m^2^) to minimize weight gain with no effect on infant size at birth. However, personalized decisions with risks and benefits (particularly long-term fetal outcome and gastrointestinal side effects) have to be discussed.

In pregnant women with PCOS, consider continuing metformin especially in those with a BMI ≥30 kg/m2, even if in this group of women, metformin also does not reduce infant size.

In pregnant women with GDM, consider metformin in very obese women who are likely to need insulin as metformin will reduce the dose needed and gestational weight gain.

In pregnant women with T2DM already on metformin, consider continuing metformin throughout pregnancy; however, stop taking metformin if there is evidence of fetus being SGA; consider initiating treatment in obese women who are insulin naïve and consider adding it to those on large dose of insulin to reduce dose.

They concluded that, owing to increasing rates of maternal obesity, GDM, and T2DM, metformin use in pregnancy is increasing; overall, it appears safe and effective but further research is needed to examine mechanisms linking metformin to obesity reported during childhood in some follow-up studies.

An interesting work was made by Tarry-Adkins et al. in a recent big meta-analysis (75). They have included 35 RCTs reporting pregnancy outcomes in women randomized to metformin versus any other treatment for any indications. The sample included 8,033 patients and the analysis showed that metformin use is associated with lower gestational weight gain and a modest reduced risk of preeclampsia, but increased gastrointestinal side effects compared to other treatments.

Metformin is safe and effective in pregnant women with insulin resistance. Currently, there remain a lot of blind spots in the use of metformin in pregnant women; some interesting clinical trials are ongoing (76), though, with the hope of providing us more clinical evidence and certainties on metformin use in this field.

## Author contributions

DP contributed to conception and design of the study. DP, AB, and GT researched and selected the articles of interest. GT and AB (that share first authorship) wrote the first draft of the manuscript and wrote sections of the manuscript. All authors (AB, GT, LT, LV, AP, SD, AR, MD, AL, DP, and AS) contributed to manuscript revision. All authors contributed to the article and approved the submitted version.
